# Behavioral and Network Origins of Wealth Inequality: Insights from a Virtual World

**DOI:** 10.1371/journal.pone.0103503

**Published:** 2014-08-25

**Authors:** Benedikt Fuchs, Stefan Thurner

**Affiliations:** 1 Section for Science of Complex Systems, Medical University of Vienna, Vienna, Austria; 2 Santa Fe Institute, Santa Fe, New Mexico, United States of America; 3 International Institute for Applied Systems Analysis, Laxenburg, Austria; University of Zaragoza, Spain

## Abstract

Almost universally, wealth is not distributed uniformly within societies or economies. Even though wealth data have been collected in various forms for centuries, the origins for the observed wealth-disparity and social inequality are not yet fully understood. Especially the impact and connections of human behavior on wealth could so far not be inferred from data. Here we study wealth data from the virtual economy of the massive multiplayer online game (MMOG) *Pardus*. This data not only contains every player's wealth at every point in time, but also all actions over a timespan of almost a decade. We find that wealth distributions in the virtual world are very similar to those in Western countries. In particular we find an approximate exponential distribution for low wealth levels and a power-law tail for high levels. The Gini index is found to be 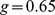, which is close to the indices of many Western countries. We find that wealth-increase rates depend on the time when players entered the game. Players that entered the game early on tend to have remarkably higher wealth-increase rates than those who joined later. Studying the players' positions within their social networks, we find that the local position in the trade network is most relevant for wealth. Wealthy people have high in- and out-degrees in the trade network, relatively low nearest-neighbor degrees, and low clustering coefficients. Wealthy players have many mutual friendships and are socially well respected by others, but spend more time on business than on socializing. Wealthy players have few personal enemies, but show animosity towards players that behave as public enemies. We find that players that are not organized within social groups are significantly poorer on average. We observe that “political” status and wealth go hand in hand.

## Introduction

The richest 1% own nearly half of all global wealth. The richest 10% claim about 86%, so that 90% of the world's population have to share the rest [1]. Wealth is not distributed evenly across nations or economies. The inequality of wealth is a strong driving force in human history and has been given much attention ever since the onset of economics. The definition of wealth is not straight forward and varies widely across history and schools of thought. Adam Smith uses the word *stock* for the personal possessions and regards everything except material goods as per se worthless [2]. Wealth is defined by Thomas R. Malthus as “those material objects which are necessary, useful, or agreeable to mankind” [3, p. 28], and by John S. Mill as “all useful or agreeable things which possess exchangeable value” [4, p. 10]. Alfred Marshall in his definition includes immaterial goods, such as personal skills, as long as they can be transferred [5]. To accumulate wealth, income must exceed the needs for immediate survival [2], [4], [5], which implies that a society living at the subsistence level is basically egalitarian, since no-one can accumulate wealth. As soon as societies produce a surplus, social stratification arises [4], [6]–[8], and universally leads to an unbalanced distribution of wealth [9].

The quantitative study of personal wealth distributions begins with Vilfredo Pareto [10], who observed that the tails of wealth distributions in a variety of datasets follow a power-law, 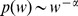. Pareto thought that this power-law appears universally across times and nations. Indeed it is found in an impressive number of data, including ancient Egypt, medieval Hungary, present day Europe, USA, Russia, India, and China [11]–[19]. We present a collection of data in Tab. S1 in the SI. For these countries the power-law exponents range from 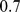 to 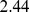. Datasets containing both the bulk of the population and the richest show a double power-law [20]: while exponents dealing with the richest, like [14]–[17], are close to (sometimes below) 1, exponents describing the bulk of the population, like [11], [12], [18] are found to be around 2. In *Pardus*, the extremely rich class is absent. The power-law exponent 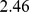 found in *Pardus* is at the high end of exponents describing the moderately rich.

Empirical data of wealth distributions is a non-trivial issue, the main difficulty being to obtain correct wealth data of individuals [5], [21]. Most countries have an income tax, only a few employ a wealth tax. Out of the 158 countries and territories listed in [22], 149 levy tax on income, only seven on wealth. Income tax data can be used to generate income distributions to study wealth-increase and re-distribution dynamics of the low- and medium income classes. Sometimes income has been used as a proxy for wealth [23]–[27], with the problematic assumption that income is approximately proportional to wealth plus human capital [28]. Income of the richest is often not reflected in income tax data, since their wealth increments are usually not related to salaries, but are usually due to capital gains. Therefore the tail of the distribution is often not seen in tax-based data: wealth distribution data poses a challenge to this day.

**Figure 1:**
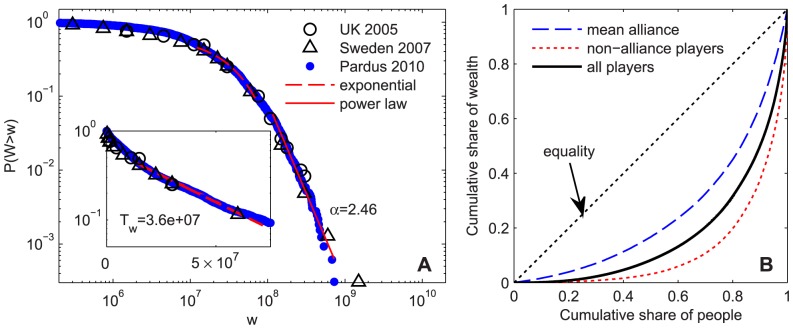
Wealth distribution and Lorenz curve. A Cumulative wealth distributions for the United Kingdom, Sweden, and for the *Pardus* MMOG on day 1200. (Data sources: [29], [31]) People with negative wealth have been excluded. A power-law tail is visible. The exponent is determined with a least-square fit to the richest 5% of the population. The bulk of the distribution, i.e. the richest 50% to 10%, can be fitted with an exponential function 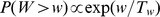 (inset). The poorest obviously do not follow an exponential distribution, while the richest 10% are the crossover region to a power-law. B Lorenz curve of wealth in *Pardus* on day 1200 (excluding newcomers and inactive players). For every alliance, a separate Lorenz curve is calculated. The dashed blue curve represents the average of these single alliance Lorenz curves.

In this work we are primarily interested in wealth distributions, and attempt for its explanation in terms of behavioral and network aspects. Data on wealth distributions is obtainable from countries imposing a tax on wealth, like Sweden [29], [30] (abolished in 2007 [22]), surveys on wealth [11], adaptions of data on inheritance tax [12], [31], the size of houses found in an excavation [13], the number of serfs from a historical almanac [14], and top-rich rankings in magazines [15]–[17]. In Fig. 1 A the wealth distributions for the UK in 2005 and Sweden in 2007 are shown. Both exhibit a power-law tail, whereas the bulk of the distribution is better described with an exponential (inset). There is evidence that in many economies the wealth distribution for low wealth levels follows an approximate exponential function [12], whereas the tail follows an approximate power-law [10]–[18], [20]. Consumption can not sustainably drop below the minimum income needed to exist. To avoid the consequences of consumption below the minimum income needed to exist, many modern countries provide welfare. This leads to the situation that a significant fraction of the population can have practically no wealth (for example 24% of Swedish households had negative or zero net wealth in 1992 [32]), but very few have income below the minimum that is needed to exist. A number of models have been suggested to understand the features of empirical wealth distributions and relate them to appropriate mechanisms. While power-law distributions can be understood by a multiplicative re-distribution processes that favors the part of the population that are wealthy enough to hold substantial financial assets, the bulk of the distribution can be understood by relatively simple exchange models. The first models to explain a power-law income distribution (in most cases the tail) were brought forward in [33]. A model incorporating both proportional growth and exchange was suggested in [34]: 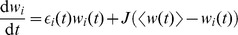. Here 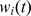 is the wealth of individual 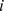 at time 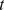, 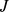 is a coupling constant and 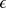 is a random variable with mean 0 and finite standard deviation 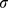 independent of 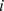 and 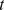. The model has a stable solution with an asymptotic power-law for large 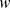 with a power exponent of 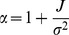. In [21], [35], a Fokker-Planck equation was proposed for the income distribution. It leads to an income distribution that behaves like an exponential for small and mid-range incomes, and as a power-law for the highest incomes. The interpolation between the bulk and the rich is different from the one in [34]. The model has been extended to capture a second power-law for the super-rich [36]. To understand the exponential distribution of the bulk, simple additive wealth exchange models can be used. For example in [37] at each time step 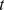, a pair of agents 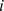 and 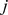 is chosen randomly, and exchange an amount 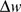 of money, so that 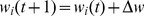, and 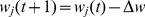. To avoid agents with infinite debt, a minimum (negative) wealth is imposed so that the exchange only takes place if 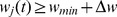. The exponential distribution has been used to describe the (bulk of the) wealth distribution of the UK and income distributions of the UK, the USA [12], and Australia [38]. Extending the additive exchange model by only allowing exchange between agents that are neighbors in a network instead of all possible pairs results in a wealth distribution that follows the degree distribution of the network, possibly generating a power-law tail [39]. A similar mechanism has been suggested for the productivity of firms instead of wealth [40]. Adding a savings propensity 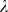 to the simple exchange model [37] means that agents use only a fraction 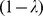 of their wealth for exchange, 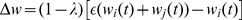. Here 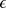 is a random variable between zero and one. This leads to a Gamma distribution of wealth 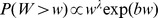 [41], with 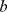 a constant. If this savings propensity is drawn from an uniform distribution over 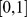, a distribution with a power-law tail follows [42]. Another model that leads to the Gamma distribution is derived from the concept of social stratification. The model is given by 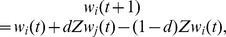 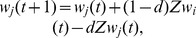 where individuals 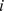 and 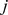 are chosen randomly at each step, 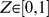 is a random variable, and 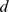 is a binary random variable, zero or one [28]. The resulting function has been used to fit income distributions of the UK and USA [25]. There are several models of multiplicative wealth growth [43], 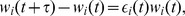 that lead to log-normal cumulative distributions, 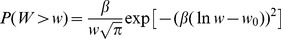. Models of this kind have been used to describe income distributions [23], [24]. Other functions that effectively interpolate between an exponential in the low wealth regime and a power-law tail, include the Tsallis distribution (*q*-exponential), 
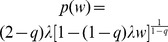
, which has been applied to the distribution of income in Japan, UK and New Zealand [26], with 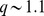. Another generalization of the exponential function, 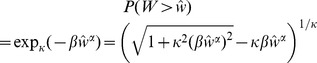, with 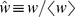, 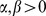, and 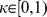, has been fitted to income distributions of Germany, Italy, UK [27], Australia, and USA [44].

It was hitherto impossible to directly study wealth of individuals as a consequence of social performance indicators, positions and roles within social networks, or behavioral patterns. However, in the context of massive multiplayer online games (MMOG) there exists an opportunity to study the origin of wealth of individuals as a function of their position within their social networks and behavioral patterns. In this paper we use data from the MMOG *Pardus*, where people live a virtual life in synthetic (computer game) worlds [45]. The essence of MMOGs is the open-ended simultaneous interaction of thousands of players in a multitude of ways, including communication, trade, and accumulation of social status. The number of “inhabitants” of some of these virtual worlds exceeds the population of small countries: *World of Warcraft*, started in 2004 and currently the biggest MMOG worldwide, has about 7.7 millions of paying subscribers as of June 2013 [46]. Production and trade between players is a common feature of many MMOGs, and can create a complex and highly structured economy within the game. Although all goods produced and traded are virtual, the economy as such is real: players invest time and effort to invent, produce, distribute, consume and dispose these virtual goods and services. Virtual goods produced in some MMOGs can be traded in the real world for real money, which then allows to measure hourly wage and gross national product of a MMOG [47]. In some MMOGs, entire characters (avatars) are traded for money in the real world, which allows to quantify “human capital”, such as skills, influence on others, leadership, etc. Economical and sociological data are easily accessible in virtual worlds in terms of log-files, and have become a natural field for research [48]–[56], even allowing economical experiments [57].

The particular dataset of the *Pardus* game comprises complete information about a virtual, but nevertheless human, society. We have complete knowledge of every action, interaction, communication, trade, location change, etc. of each of the 40,785 players at the time resolution of one second. The society of the *Pardus* game has been studied extensively over the past years. Social networks have been quantified with respect to their structure and dynamics, revealing network densification [58], corroborating the “weak ties hypothesis”, and showing evidence for triadic closure as driving mechanism for the evolution of the socially positive networks [50], [51]. The empirical multiplex nature of the social networks allows to quantify correlations between socially positive interactions, and between various types of interactions [52]. Mobility of avatars, as studied within the *Pardus* world, shows striking similarities to human travel in the real world [53]. Timeseries of actions in the *Pardus* game have been used to quantify the origin of good and cooperative behavior. It has been attempted to predict actions of avatars, given the information of their past actions in [54]. Social network formation dynamics within *Pardus* has been used to demonstrate the existence of gender differences in the social networking behavior of male and female avatars [55].

### The MMOG *Pardus*

The MMOG *Pardus* provides a persistent synthetic world in which thousands of players interact through their game characters (avatars) which they control through their browser. Players tend to identify with their characters [45], which allows us to write “player” for “the player's character” in the following. The setting of *Pardus* is futuristic. Every player owns a space ship to travel the “universe”, which contains planets, space cities, natural resource fields, and even space monsters. Players can explore the universe, build production sites (factories), trade with each other, and fight each other, or against monsters. Many players are driven by the accumulation of “social status” by obtaining honors for certain social achievements or by purchasing expensive items that serve as status symbols. There is no overall goal in the game, and players constantly define their own goals and roles. *Pardus* is free of charge but requires a registration. In total, more than 400,000 players have registered since 2004. *Pardus* has an internal “unit of time” called *Action Points* (AP). At every day every player has a limited number of 5000 APs that can be spent. Different actions of the player “cost” various amounts of APs.

#### The economy of *Pardus*

The input-output production matrix of the economy and the variety of goods are pre-defined within the *Pardus* framework. Goods are of uniform quality (homogeneous). Consumables and equipment can be partially substituted by other types of consumables and equipment, while intermediate goods are needed for production in exact proportions. There are five commodities that are natural resources, 19 serve as intermediate goods, and five are end-products, i.e. consumables.

Although capital requirements to create production facilities are low, there are barriers to entering production. Incumbents may threaten or harm potential new entrepreneurs. Game rules set an upper limit of production facilities for every single player. Many players operate the maximum number of factories. Production facilities in *Pardus* are fixed assets with infinite durability but can not be sold. Investments into production facilities therefore motivate incumbents to stay in the sector (exit barrier). While no labor is needed for production itself, transport of raw materials and intermediate goods requires effort and resources of the players. Because of transport costs, facilities effectively only compete with similar facilities which are close by. Together with sparse distribution of production facilities, this leads to effective local oligopolies.

A special kind of goods are various forms of equipment, i.e. items like space ships or weapons. Equipment can only be produced in special facilities which also act as warehouses and selling points. Equipment is durable, but has a finite lifetime. Maintenance applied to equipment can increase the lifetime. When a player sells equipment after usage, it is scrapped. (Players can own only a limited amount of equipment, resulting in an incentive to sell from time to time.) Owners of production facilities are completely free to set the price at which they buy their raw materials and sell their products. There are also non-player facilities (belonging to the game itself) whose prices directly react to local supply and demand within certain limits. The monetary currency of *Pardus* is called *credits*. There is no banking system and all transactions are payed and cleared immediately. There is no inflation in the game.

#### The social groups in *Pardus*

Players can organize themselves within social groups for various purposes. Groups often share the same interests, or are constituted as pirate groups, exploration teams, self-defense units, etc. Usually groups do not get larger than approximately 140 members. *Pardus* provides administration tools for officially declared groups, which are then called *alliances*. Alliances have a common cash pool which they use for their goals, like defense or production. Often alliances are created and used for economic purposes. Alliances can locally coordinate production capacities to build up entire production chains. For an optimal production chain, it is sometimes necessary to increase the production capacity of a certain intermediate good. This is often done by luring a new member into the corresponding business, and by paying her for the construction of an additional production facility.

#### Wealth of players

There are several ways for players to obtain wealth: trading, collecting natural resources, producing goods, working for hire (most common jobs are courier/teamster, hunter, or bounty hunter), receiving donations or other payments, an increase of the alliance funds (by payments from someone else), and robbing or stealing.

We define wealth or “personal net-worth” of player 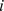 as the sum of the value of his assets, i.e. liquidity (cash) 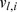, equipment 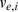, share of alliance funds 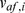, and inventory 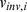. The latter are the commodities that are stored in player 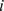
's production facilities and in the space ship. Equipment are various in-game items like a space ship or weapons. Each type of equipment can be bought (new) at varying prices and sold (used) at a constant price. At non-player facilities, equipment can be bought for twice the sell price. To determine the contribution to the net-worth, we therefore take 1.5 times the listed sale price as the current “value” of each piece of equipment. The values of the different types of equipment span five orders of magnitude. The share of the alliance funds, if the player is a member of an official group, is calculated by evenly dividing the group's cash pool to all members. Additionally, it is discounted by a factor of two. Inventory is neglected, an exception being those warehouses that are associated with the production of equipment. Real estate, i.e. the production facilities, can not be sold and therefore has no market value.

There are several ways to reduce wealth in the game: consuming, paying for maintenance (either because of “natural” degradation or because of damage from a fight), investing into production facilities or equipment, discarding goods, becoming victim of theft or robbery, giving to fellow players or paying into the alliance funds, a decrease of the alliance funds, or making an adverse trade. In summary, the wealth of individual 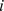 at time 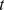 is given by:
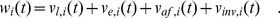
(1)


In the following we use a series of measures that are necessary to quantify wealth and performance of the avatars. To quantify *wealth* we use 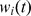 for the momentary wealth of player 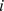 at time 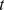. The *age* of a player is the number of days since the player entered the game for the first time. We measure the cumulative activity of a player by the total amount of APs he has “spent”. We denote this cumulative total activity by 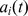. The *wealth-gain* of player 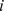 we denote by:
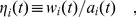
(2)which is measured in credits per AP. 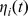 can also be seen as efficiency at gaining wealth.

There are a number of achievement-factors in the game that measure certain properties of players. The efficiency harvesting natural resources is quantified (as a game feature) by the *farming skill* of a player. Other performance related measures that players can gain and lose over time, are the *combat skill* that quantifies fighting skills, and the *experience points* (XPs), which keep a record of *fighting experience* and other activities. Players may choose to be member of a “political” *faction*, which sometimes engage in large-scale conflict (war) against each other. The *faction rank* is a measure of influence in one's faction: above a certain threshold, it grants the privilege to take part in the decision on war or peace. It is attained through several specific activities. Some players regard high combat skill, faction rank, or XP as their main goals in *Pardus*.

## Results

### The wealth distribution

Figure 1 A shows the wealth distribution of *Pardus* in comparison to the UK and Sweden. From the *Pardus* data, new and inactive players have been excluded, see Methods. The bulk of the (cumulative) distribution is compatible with an exponential [12] with decay 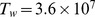 credits. The tail is best fitted with a power-law with exponent 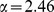. Figure S1 in the SI shows that the wealth distribution of Sweden is described well by an exponential bulk and a power-law tail. For comparison Tab. S1 in the SI contains power exponents for several “real” countries. Figure 1 B shows the Lorenz curve (see Methods) for the *Pardus* society (black line). The closer the Lorenz curve is to the diagonal (black dotted line) the more homogenous is the wealth distribution. Uniform wealth distribution corresponds to the diagonal. Associated to this line is a Gini index [59] of 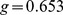. For comparison with “real” countries see Tab. 1. We further show the Lorenz curve for all players that are not organized in any alliance (red dotted line). These players generally operate individually, and show a much more pronounced wealth inequality than the entire society, the respective Gini index being 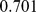. In contrast, the Lorenz curve for the various alliances (the average over all alliances with at least 5 members is shown as a dashed blue line) indicates that people within the alliances tend to be much more equal in wealth, when compared to the entire society. The Gini index for the alliances is 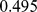. The main reason for this higher equality is the smaller fraction of poor players in alliances: while 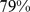 of the total population and 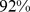 of the richest 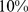 are alliance members, only 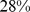 of the poorest 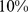 are.

**Table 1:** Measures of wealth-inequality in *Pardus* compared to real-world countries.

country	year	unit	Gini index 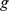	bottom 50%	top 10%	top 1%
*Pardus*	2010	all players	0.653	8.2	49.9	12.4
*Pardus*	2010	alliance players	0.495	16.7	35.4	4.6
*Pardus*	2010	Non-alliance players	0.701	3.1	62.3	20.2
China	2002	individual	0.550	14.4	41.4	–
France	1994	adult	0.730	–	61.0	21.3
Germany	1998	household	0.667	3.9	44.4	–
UK	2000	adult	0.697	5.0	56.0	23.0
USA	2001	family	0.801	2.8	69.8	32.7

Gini index 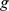, and fraction of total wealth in % held by a fraction of the population. Real-world data is taken from [61].

#### Evolution of the wealth distribution over time

**Figure 2:**
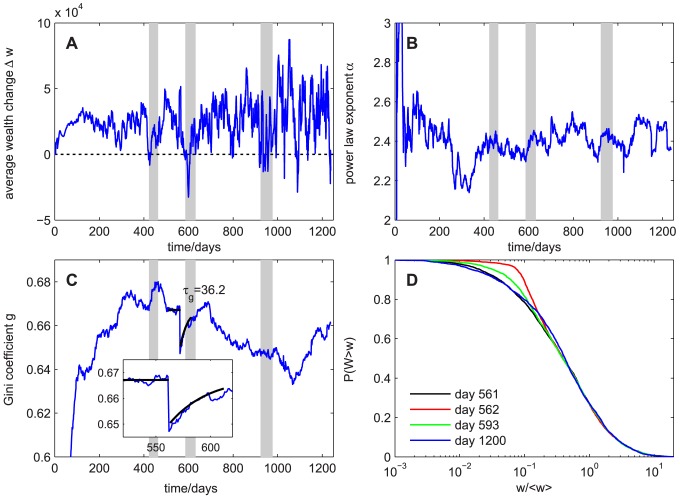
Time evolution of the wealth distribution in the entire society. A Seven day moving average of the change of the average wealth 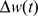. B Power-law exponent 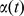, C Gini index 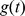. D Scaled wealth distribution at four different days (rescaled by average wealth). Gray shaded areas indicate periods of large scale war in the game. A “Christmas charity” event on day 562 led to a re-distribution from the wealthy to the poor, resulting in a downward jump of the Gini index. The inset shows the exponential recovery to the previous level.

The average wealth in the *Pardus* society 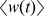 grows over time. Brackets indicate the average over all players present at time 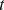. The daily average change we denote by 
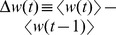
, and is presented in Fig. 2 A. We find that 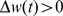 on 83% of all days, and that the average daily increase of average wealth is 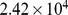 credits. In Fig. 2 A it is visible that average wealth increases less during war periods (gray shaded areas): the average daily increase during the three war periods is 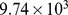, 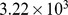, and 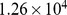 credits respectively, against 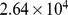 credits in peace times. Figure 2 B shows the evolution of the power-law exponent 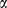. Its value is limited to a region between 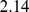 and 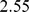. After an initial steep rise in the first 150 days, the Gini index 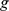 fluctuates between a maximum of 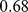 and a minimum of 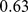, as seen in Fig. 2 C. A prominent feature is a sharp drop of 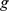 from 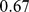 to 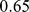 on day 562 which corresponds to 2008/12/24. At this day, a “global” charity event took place, where thousands of players donated cash for the less wealthy. The inset indicates an exponential recovery, 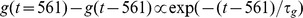 with decay time 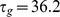 days, (black line). This indicates a remarkable stability of the shape of the wealth distribution, as also seen in Fig. 2 D: First, after dividing wealth by the average wealth on the corresponding day, the distributions on two days which are more than 1.5 years apart are very similar, see black curve (day 561) and blue curve (day 1200, identical to Fig. 1 A). Second, after a significant perturbation on day 562 (red curve, after voluntary re-distribution of wealth from the rich to the poor as “Christmas charity”), the distribution quickly returns to its previous form (green curve: one month after the redistribution). Comparing the wealth distribution on various days by the Kolmogorov-Smirnov statistic and the Jensen-Shannon divergence, we find a relaxation time of about 16 days, see Fig. S2 in the SI.

For the timeseries of 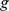 and 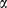 we find clear anti-correlation, with a Pearson correlation of 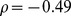 (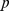 value 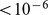, ignoring the transient phase in the first 200 days and 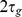 after the re-distribution). The tail of the distribution is neither affected by the charity re-distribution event nor by wars. An inverse relation for the Gini coefficient and the power-law exponent has also been observed for income in the USA [21], and is expected to a certain extent. The data from Tab. 1 [21] yield 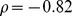 with a 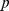 value 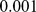. Decreasing 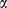 means a more pronounced tail in the wealth distribution, i.e. more extremely rich individuals, resulting in higher inequality, and therefore a higher 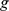.

### Individual behavioral factors for wealth

#### Influence of total activity on wealth

**Figure 3:**
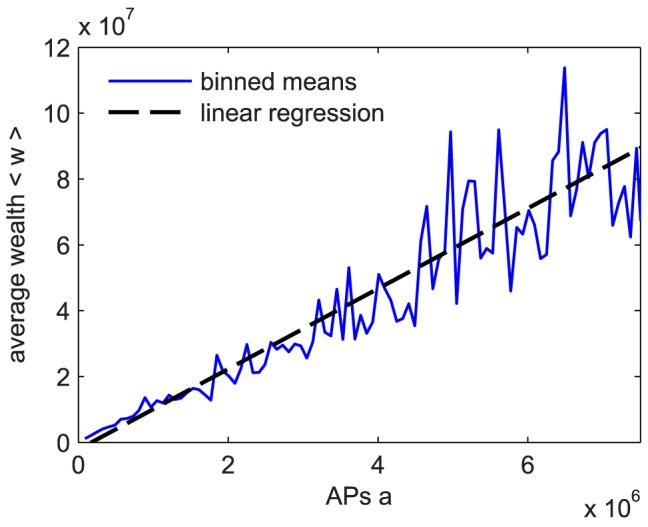
Wealth as a function of activity 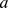. The blue line shows binned averages of wealth. The dashed line is a linear regression, with a slope 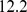 credits/AP and a corresponding Pearson correlation coefficient 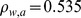. Data are taken for all active players on day 1200.

**Figure 4:**
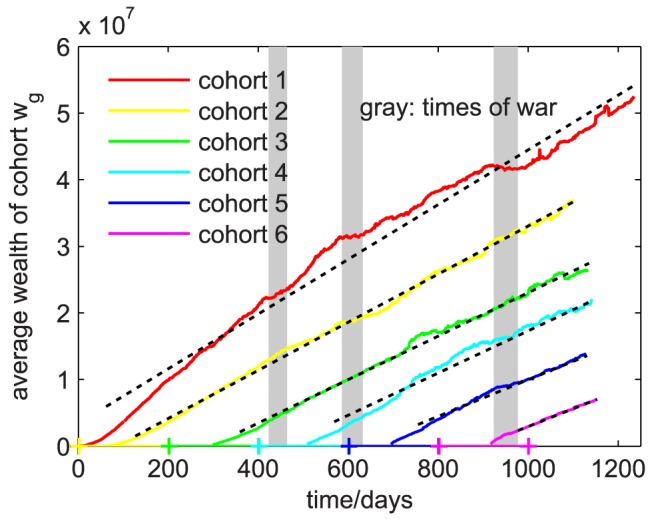
Cohort wealth as a function of time. Cohort 1 (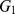) contains all players who joined *Pardus* on the first day. Cohort 2 (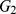) contains all players you joined between day 2 and 200, cohort 3 (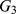) all who joined between day 201 and 400, etc. Wealth 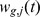 of cohort 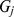 at time 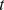 is calculated as 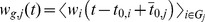, where 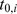 is the date at which player 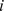 joined the game and 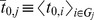 is the average cohort entry time. Gray areas mark times of war, dashed lines represent linear fits omitting the transient first 60 days.

We find a trivial strong linear relation between the average wealth of a player and her total activity, 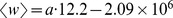, see Fig. 3. The corresponding Pearson correlation coefficient is 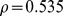 (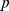 value 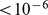). Figure 4 shows the wealth timeseries of six cohorts of players that joined *Pardus* during six different time periods. Cohort 1 contains all players who joined on the first day, cohort 2 joined between day 2 and day 200, cohort 3 between day 201 and 400, etc. For each cohort we computed its average wealth from the individual wealth timeseries of its members. For the sample, all players present on day 1238 were used. Following a short initial phase, average wealth increases almost linearly. Linear wealth-increase means that players do on average not get better at gaining wealth, i.e. they do not learn over time how to increase their wealth faster. It is also not consistent with wealth increments proportional to wealth as assumed by the Gibrat model, which would instead lead to an exponential wealth-increase on average. The slopes (i.e. wealth-increase rates) for the different cohorts are different. We find these slopes to be 4.1, 3.6, 3.3, 3.2, 2.8, and 2.7 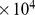 for cohort 1 to cohort 6, respectively. We used a linear fit omitting the first 60 days of each timeseries. This means that the older the cohorts, the faster is their average wealth-gain. There are two possible interpretations of this result. Either only the players that are more efficient in accumulating wealth have stayed in the game to become the old cohorts, or older players occupy the most profitable trades, locations in the game, and younger players have no chance to enter these market positions. We have checked the first interpretation by including all players up to the end of their lifetime, irrespective of whether or not they were present on day 1238, and found only a marginal effect, see Fig. S3 in the SI. However, there is a relation between wealth and lifetime: the richer a player is, the lower the probability that he will leave the game, see Fig. S4 in the SI. Effects of war on wealth can be seen. The wealth of various cohorts stagnates during war and sometimes continues to grow with a slightly different slope than before. For the younger cohorts, this effect is washed out due to their broad range of entry dates.

#### Wealth and the actions of players

Players can interact with each other through trading with each others selling points, communicating, directly exchanging goods (making gifts), attacking, placing bounties, marking each other as friend or enemy, or removing one of these marks. (The direct exchange of goods is called “trade” in [54], [55] and is treated as the same as trade in [50]) Trading, communicating, making gifts, friendship marking, or removing an enemy mark are seen as “cooperative” or “good” actions, while the remaining interactions are destructive or “bad”. For every player 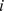 who is active on day 1200, we count the actions performed since day 1170: the number of trades he initiated 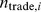, the number of messages he sent 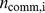, the number of gifts he made 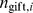, the number of attacks he did 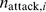, the number of bounties he placed 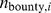, the number of other players he marks as friend 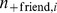 or enemy 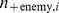, the number of friend or enemy marks he removes, 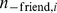 or 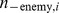. The total number of activities follows as 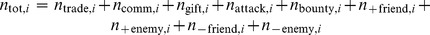. We only consider players with 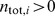. For those we define the fraction of one type of action as
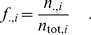
(3)


Accordingly, we define the fraction of “good” actions as
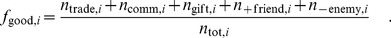
(4)


**Figure 5:**
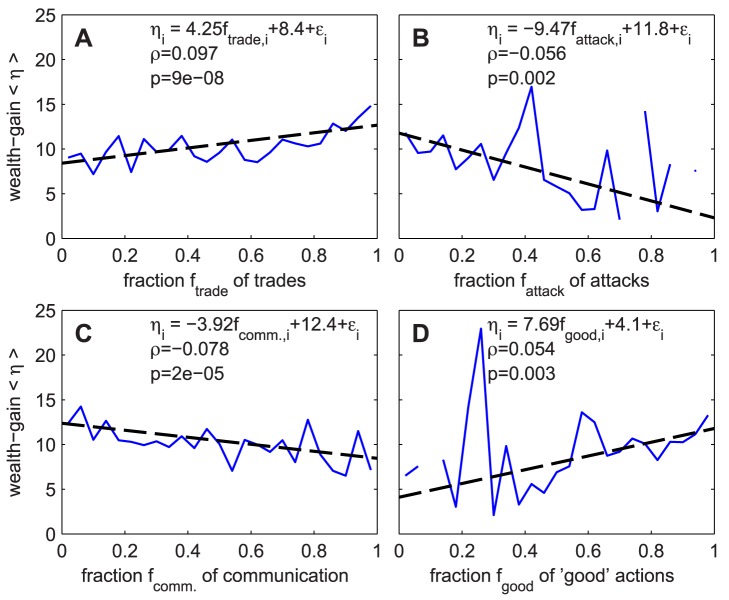
Wealth-gain as a function of behavior. Behavior is quantified as the fraction of particular actions, such as A trades, B attacks, and C communication, with respect to all actions performed by an individual. The blue line shows binned averages, the dashed line is the linear regression. Data consist of all actions between day 1170 and 1200 and wealth-gain on day 1200.

Figure 5 A clearly shows that the more a player trades compared to his other actions, the higher is his wealth-gain. This is no surprise, as trade is the main source of income in the game. The average fraction of trade is 75.3%. Table 2 also shows a strong positive partial correlation (see Methods) between trade fraction and wealth. Figure 5 B shows that the more of a player's actions are attacks, the lower is his wealth-gain. This suggests that revenue from attacks through robbery and bounty hunting does hardly or not exceed the costs for repairing damage done by the fight. There might be secondary damaging effects of aggressive behavior, such as reduced willingness of others for trade. A third explanation might be that attacks are sometimes carried out without the intent to rob or to collect a bounty, but just for terror. The average fraction of attacks is 1.7%. Table 2 shows a significant negative partial correlation between attacks and wealth. It can be seen in Fig. 5 C that players who communicate much have lower wealth-gains. The main reason for this might be that if a high fraction of the actions consists of sending messages, only a low fraction of actions consists of trades: while trades are directly influencing wealth, communication is neutral. Of course, the same also applies to attacks. The average fraction of communication is 19.7%. Table 2 shows a significant negative partial correlation between communication and wealth. Figure 5 D shows that a higher fraction of “good” actions is connected to higher wealth-gain. The average fraction of “good” actions is 97.5%. “Good” actions are mainly trades (which are on average 3/4 of *all* actions and therefore an even higher fraction of “good” actions), while “bad” actions are mainly attacks (on average, 2.5% of all actions are “bad” while 1.7% of all actions are attacks). Therefore, high 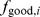 means high 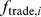 and low 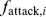, both of which are connected to high wealth-gain. The connection between high fraction of “good” actions and high wealth-gain is a direct consequence. The partial correlation in Tab. 2 also clearly shows a positive partial correlation between the fraction of “good” actions and wealth.

**Table 2:** Partial correlation coefficients for wealth controlling for total activity.

	day 240	day 480	day 720	day 960	day 1200
Age	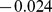	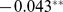	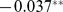	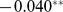	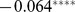
faction rank	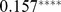	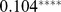	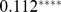	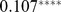	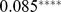
XP	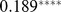	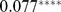	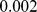	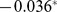	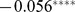
combat skill	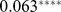	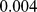	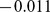	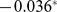	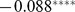
farming skill	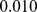	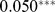	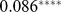	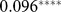	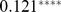
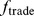	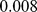	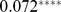	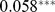	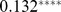	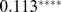
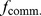	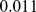	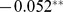	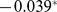	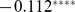	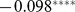
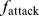	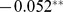	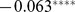	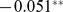	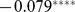	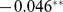
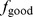	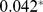	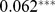	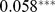	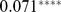	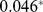
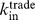	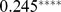	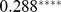	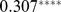	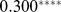	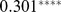
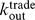	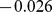	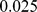	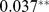	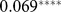	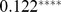
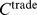	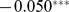	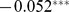	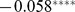	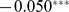	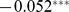
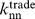	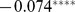	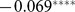	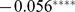	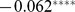	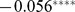
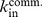	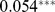	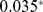	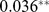	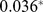	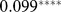
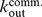	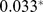	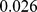	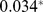	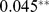	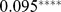
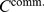	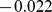	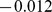	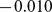	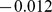	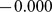
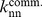	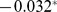	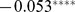	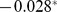	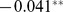	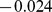
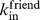	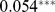	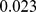	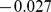	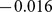	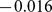
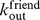	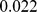	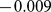	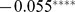	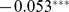	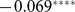
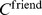	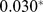	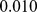	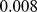	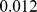	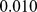
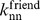	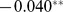	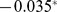	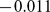	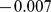	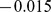
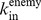	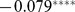	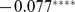	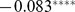	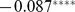	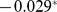
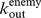	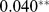	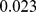	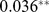	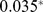	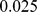
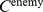	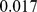	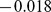	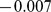	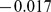	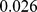
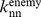	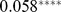	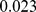	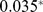	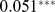	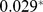

Data taken at days 240, 480, 720, 960, and 1200 after the beginning of the game. 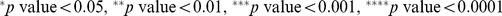.

#### Influence of achievement-factors on wealth

**Figure 6:**
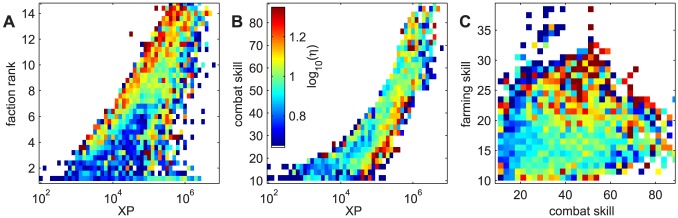
2D binned averages of the wealth-gain as a function of achievement-factors. Colors represent the logarithm of the average wealth-gain, 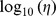, over all players that fall into that bin. Blue corresponds to low, red to high values, empty bins are white. A XP and faction rank, B XP and combat skill, C combat skill and farming skill. Data are taken every 240 days (see Methods).

Wealth as well as the achievement-factors, such as skills, XPs, and faction rank, are strongly correlated with total activity. To exclude these spurious correlations, partial correlation coefficients are calculated (see Methods) and reported in the upper part of Tab. 2. We take snapshots of the achievement-factors and of the wealth on days 240, 480, 720, 960, and 1200 respectively. Stars in Tab. 2 indicate the significance level for the null-hypothesis that the given coefficient is zero. A correlation model over all variables (see Tab. S2 in the SI) does not yield improvement over the one-dimensional regression of 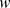 on 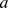 as shown in Fig. 3. To examine possible nonlinear relations between achievement-factors and wealth, in Fig. 6 we show two-dimensional binned averages of wealth-gains as a function of faction rank, XP, combat-, and farming skill. To produce two-dimensional binned averages, players were sorted into bins according to two achievement-factors. For every bin the average wealth-gain 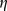 (see Eq. 2) of all players in that bin is determined and represented as the color of the bin. If no player with a certain combination of achievement-factors is found, the corresponding bin is empty, and the bin color is white.

From Fig. 6 and Tab. 2 we find the influence and significance of the various factors:

**Age** is a significant factor (significance level below 1%) at four out of the five time points. The negative coefficient seems to be in contrast to the increase of wealth with age seen in Fig. 4. The explanation is that total activity, which is most strongly correlated with wealth, is limited by age. This induces the spurious correlation between wealth and age seen in Fig. 4.

**Faction rank** is a significant positive factor for wealth with a significance level below 0.01% for all days. High faction rank means “political” influence in the game. Players that are in no faction, i.e. less social, have the smallest possible value as faction rank and are on average poorer. Figure 6 A shows that a high faction rank correlates strongly with wealth-gain. We also see that the non-empty bins suggest a strong correlation between XP and faction rank.

**XP** is significantly positive for wealth on the first two sample days with continually decreasing coefficient, changing sign on the last two days. This might indicate that XP is positive up to a certain extent, after which the goal of high XP starts to contradict the goal of high wealth. In Fig. 6 A, the data from all five days are combined, and the positive and negative correlations of XP cancel and leave no significant effect of XP on wealth-gains.

**Combat skill** has a correlation with wealth similar to XP, see Tab. 2. We see in Fig. 6 B that combat skill is approximately proportional to the logarithm of XP. There is a significant fraction of rich people with low combat skill of about 20. Figure 6 C shows no correlation between combat skill and wealth-gains.

**Farming skill** has a consistently positive and mostly significant correlation with wealth. Farming skill is associated with the collection of resources, which generates income. Figure 6 C also suggests an association between high farming skill and high wealth-gains.

### The effects of groups on wealth: the value of being social

**Figure 7:**
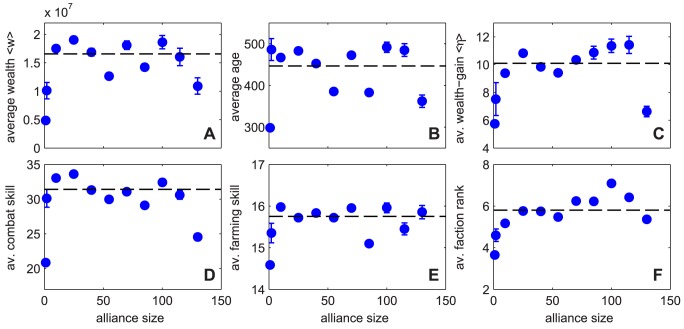
Wealth and other properties as a function of alliance size. We bin players according to the size of the alliance they belong to, and show various properties as a function of alliance size: A wealth, B age, C wealth-gain, D combat skill, E farming skill, F faction rank. First bin are players in no alliance, second bin are players in an alliance of size two. Clearly members of these smallest alliances show low wealth and achievement measures. Also for the largest groups, lower levels are observable. Error bars denote the standard errors of these means (assuming Gaussian distributions). The black dashed line shows the average over all players in an alliance with at least three members. Data are taken every 240 days (see Methods).

Players in *Pardus* organize within social groups that are called alliances. At day 1200, 161 alliances with an average size of 23 members existed. Being a member of an alliance is a social commitment. In Tab. 3 we collect the average values for several features of players, depending on whether they are alliance members or not. In general, alliance members are richer, both in absolute terms and in terms of wealth-gains than non alliance members. Members also have better skills and a higher faction rank. In Fig. 7 we see that the size of an alliance has little influence on wealth and other factors, except for players that are in alliances with only two members. These are consistently poorer than the players in groups with three or more members. Members of the biggest alliances also have some indicators below average (dashed line).

**Table 3:** Various properties of players versus their membership in alliances.

Average	no alliance	alliance
wealth 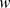	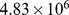	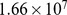
age	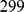	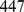
total activity 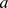	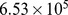	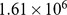
wealth-gain 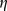	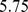	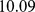
combat skill	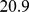	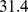
farming skill	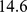	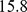
faction rank	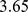	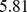

All 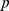 values obtained from a two sample 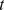-test and a Wilcoxon rank sum test are less than 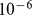. Data are taken every 240 days (see Methods).

### The effects of social networks on wealth

**Figure 8:**
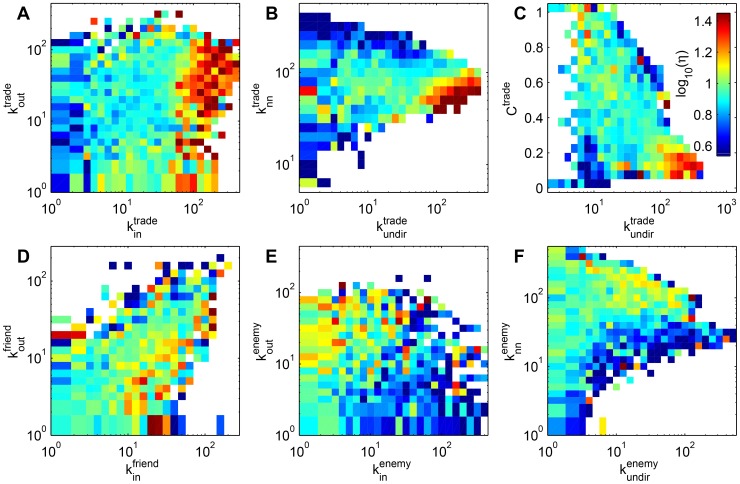
Wealth-gain as a function of network properties. Color represents the logarithm of the wealth-gain, 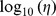, from blue (lowest) to red (highest), empty bins are white. A trade in- and out-degree, B trade undirected degree and nearest-neighbor degree, C trade undirected degree and clustering coefficient, D friend in- and out-degree, E enmity in- and out-degree, F enmity undirected degree and nearest-neighbor degree. Data are taken every 240 days (see Methods).

We use the trade, communication, friendship, and enemy networks of *Pardus* (see SI), which are available for every day. For every node (player) 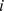 we determine the in- and out-degree (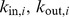), the nearest-neighbor degree 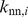, and its clustering coefficient (see SI). We calculate partial correlations between wealth and the network parameters controlling for total activity. We collect the results in the lower part of Tab. 2. To elucidate the dependence of wealth on various combinations of network factors, in Fig. 8 we plot two-dimensional binned averages of wealth-gain versus pairs of network properties. The results are:

#### Trade network

As expected, the trade network has the strongest influence on wealth. Trade in-degree has a significant, positive partial correlation with wealth. The in-degree is defined as trade with a player's production facilities and is therefore a proxy for his production. Figure 8 A confirms the positive connection between trade in-degree and wealth, while not showing any influence of trade out-degree. However, Tab. 2 reports a positive correlation between wealth and active trade with the production facilities of fellow players, in agreement with the positive effect of active trading shown in Fig. 5 A. Figure 8 B presents the undirected degree of the trade network versus the nearest-neighbor degree. The richest are found to have an intermediate nearest-neighbor degree of about 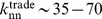, well below their undirected degree. This means that they are selling to people that are less connected in the trade network than they are themselves. Table 2 shows a negative correlation between the nearest-neighbor degree and wealth with a significance level below 0.01%. From Fig. 8 C we gather that high wealth-gain is made with a combination of high degree and a relatively low clustering coefficient, 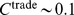. This means that rich players avoid cyclical structures in their trading networks, which allows them to act as “brokers” between players that do not directly trade with each other. The partial correlation coefficient between wealth and the trade clustering coefficient is negative.

#### Communication network

Communication in-degree has a significantly positive partial correlation coefficient. High communication in-degree means good access to information, which is expected to be profitable. The Communication out-degree shows positive partial correlation on most days. A player's communication out-degree is the number of fellow players she *tries* to influence. Since most communication links are reciprocal, and in- and out-degree are therefore highly correlated, there might be a spurious effect of the communication in-degree. The communication nearest-neighbor degree has a negative and mostly significant partial correlation coefficient. This might indicate it is advantageous to mainly converse with fellow players who are less informed than oneself.

#### Friendship network

In Fig. 8 D the situation for the in- and out-degrees for the friendship network is shown. It is visible that players with high wealth-gain are those that are liked by more players than they like themselves, 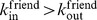. Poor players have marked fellow players as friends more often on average than they have been marked. In Tab. 2, friendship in-degree hardly shows any correlation with wealth, while friendship out-degree has a significant negative correlation on all sampling days except day 240. This might indicate that time and resources invested into friendship are missing for the generation of wealth.

#### Enmity network

We see that people with above average wealth-gain are very rarely marked as an enemy by others, but do mark others as enemies, see Fig. 8 E. Players who have been marked as enemy by many others are generally poor. In agreement with this finding, the enmity in-degree has a significant negative partial correlation with wealth, while the enmity out-degree has a weak significant positive correlation with wealth, see Tab. 2. This suggests that players with high wealth-gain actively invest in a good reputation. Finally, players with above average wealth-gain have a high nearest-neighbor degree, see Fig. 8 F. Table 2 reports (mainly) significant positive correlations between wealth and the enmity nearest-neighbor degree. Players with high enmity (in)degree are “public enemies” [50]. A high 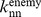 means that one is mainly the enemy of public enemies and that one has few private enemies.

## Discussion

We studied the economy of the virtual world of the MMOG *Pardus*. We found that the wealth distribution in *Pardus* has a similar shape like wealth distributions of “real” economies, including an exponential bulk and a power-law tail. The power-law exponent of *Pardus* is within the range of real-world power-law exponents describing the moderately rich. The Gini index shows that wealth is slightly more equally distributed in *Pardus* than in many Western industrial countries. We observe that the shape of the wealth distribution is stable: eventual external perturbations exponentially relax to a stationary state. While the total wealth in *Pardus* increases over time, large scale conflicts hamper the creation of wealth. We found that an average player's wealth grows linearly with his total activity. As total activity is limited by a player's age (time in the game), wealth also increases linearly with the age of a player. Linear increase suggests that neither learning nor proportional growth (i.e. “rich get richer”) are dominant on a global scale. Players who entered the game earlier have higher wealth-increase rates.

For the first time, we could observe the connections between personal wealth and social behavior. We found that wealthy players organize in social groups. A group size between three and 120 members appeared to be best for wealth and achievement-factors. We found that wealthy players invest in their social reputation by constructive actions. Personal wealth in *Pardus* is connected to skills for collecting resources and high “political” influence, but not to combat skill and fighting experience. Analyzing the trade network, we observed that wealthy players trade with many others, while their trade partners trade with fewer others, and hardly among each other. Taken to the extreme, the wealthy organize their local trade network so that they are the hub of a star-like network. In the friendship and enmity networks we observed that the wealthy are well respected, and show animosity – if at all – only towards public enemies.

## Materials and Methods

### Datasets

We study data from one of three game universes of *Pardus*, *Artemis*. Days are counted from the opening of this server, day one is June 12, 2007. For dataset 1, used for Figs. 1, 3, and 5, we extract snapshot data on day 1200 since the opening of the game, i.e. September 23, 2010. We selected only those players that have been active in the last 30 days before day 1200. For dataset 2, used for Fig. 2, we extracted data on every day and applied the same filtering as for dataset 1, i.e. we excluded players whose last activity was longer than 30 days ago. For dataset 3, used for Fig. 4, we took the complete timeseries of all players that were in the game on day 1238, which is the last day included in our database. For dataset 4, used for Figs. 8 and 7 and Tabs. 2 and 3, we used snapshot datasets separated by 240 days, starting at day 240. After 240 days, the autocorrelation function of wealth has decayed to 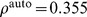, so the single data points can be treated as independent. The data contain a daily snapshot of the friendship and enmity networks, all players' possessions, and alliance membership. For the trade network, we draw a link on day 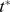 if a trade has taken place within the time range 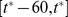. Players who have only recently joined the game are naturally close to their initial wealth and are therefore excluded from datasets 1, 2, and 4. As a criterion for admitting a player to the dataset, we require that the players have actively played for ten days, or more precisely that they have spent at least 50,000 APs. Dataset 1 contains 3,245 players, dataset 2 contains 4,483,175 data points from 16,662 distinct players, dataset 3 contains 3,693 players, and dataset 4 contains 25,195 data points from 12,186 distinct players on 5 distinct days. Dataset 1 is a proper subset of dataset 2, and also of dataset 4.

The power-law shown in Fig. 1 A was determined by a linear least-square fit to the logarithms: For 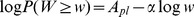, 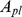 and 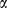 were determined minimizing 
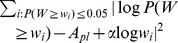
. In a similar way the exponential in Fig. 1 A was obtained by choosing 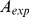 and 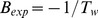 minimizing 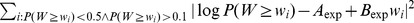.

### Lorenz curve and Gini index

Let 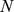 be the number of players, and 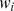 the wealth of player 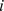, ordered so that 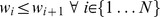. The Lorenz curve is given by the coordinates
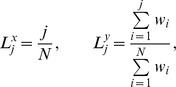
and their piecewise linear connection. For complete equality, 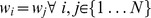, 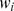 cancels and 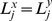, turning the Lorenz curve into a straight line from 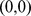 to 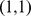.

Let 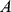 be the area under the Lorenz curve. The Gini index [59] is defined as 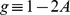. It can be calculated from the data by
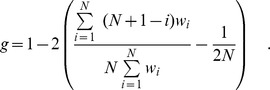



For complete equality, 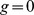, and for maximal inequality (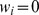 for 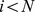), 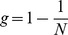.

### Correlation coefficients and partial correlations

Throughout the paper we report correlations by the widely used Pearson's correlation coefficient, calculated from data as [60]

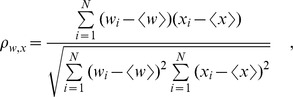
where 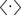 denotes the average over all 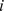. To determine the effect of single factors on wealth while removing the effect of total activity, we calculate *partial correlations* controlling for total activity: For both wealth 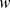 and the studied factor 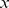, the linear regression on total activity 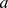 is calculated. The correlation between residuals of these regressions is the partial correlation coefficient 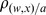 [60]. Equivalently, 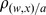 can more easily be calculated as
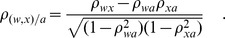



## Supporting Information

Figure S1 **Wealth distribution of Sweden from 1999 to 2006 with fits.** Black triangles mark the data, the continuous blue line is a power law fit with exponent as indicated, and the broken blue line is an exponential fit with “wealth temperature” as indicated. Data source: [30].
(EPS)

Figure S2
**Comparison between renormalized wealth distribution on day 1200 and on a varying second day.** Wealth is rescaled by the daily mean wealth 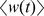, The rescaled distributions are compared by A the Jensen-Shannon divergence and B the Kolmogorov-Smirnov statistic. [60]. Black curves fit the decay of the perturbation by an exponential with decay time A 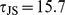 and B 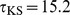. Dotted black lines mark the previous level.(EPS)

Figure S3
**Cohort wealth as a function of time.** Cohort 1 (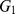) contains all players who joined *Pardus* on the first day. Cohort 2 (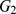) contains all players you joined between day 2 and 200, cohort 3 (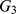) all who joined between day 201 and 400, etc. Wealth 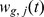 of cohort 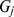 at time 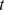 is calculated as 
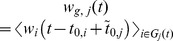
, where 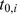 is the date at which player 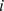 joined the game and 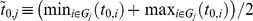 is the average cohort entry time. Players are considered as long as they are in the game, i.e. the sizes of the cohorts are not fixed, but may decrease over time, 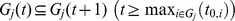. Gray areas mark times of war, dashed lines represent linear fits omitting the transient first 120 days. The slopes of these lines are 4.3, 3.5, 3.4, 3.6, 3.3, and 3.2 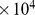 for cohort 1 to cohort 6, respectively.(EPS)

Figure S4
**Probability to leave the game as function of age and wealth.** Every day except the last, players are sorted into bins according to their current age and their current wealth: 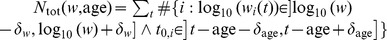, where the 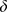
s denote half the bin size (and all other quantities as in the caption of Fig. S3). In a similar way, we count players per bin that are not in the game anymore the next day, 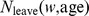. The frequency (empirical probability) for a player with a certain combination of wealth and age to leave the game is than 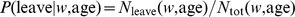. Color in the plot denotes 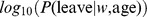, bins with insufficient data, i.e. 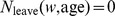, are colored white. Only players who had “spent” at least 50,000 APs have been taken into account.(EPS)

Table S1
**Comparison of power law exponent** 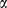 **of the** ***Pardus*** **wealth distribution to real world data.**
(PDF)

Table S2
**Linear regression model for wealth.** Data taken at days 240, 480, 720, 960, and 1200 after the beginning of the game. 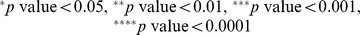.(PDF)

Data S1 **Data as plotted in** **Figs. 1** **–** **8** **.**
(ZIP)

Text S1 **Definition of Network Properties.**
(PDF)
