# Supplementary material for: Behavioral and Network Origins of Wealth Inequality: Insights from a Virtual World
Source: PLoS One. 2014 Aug 25;9(8):e103503. doi: 10.1371/journal.pone.0103503 (PMC4143195; doi:10.1371/journal.pone.0103503)
Supplement: Table S1 — Comparison of power law exponent of the Pardus wealth distribution to real world data. (PDF) [file pone.0103503.s005.pdf]

Table S1. Comparison of power law exponent α of the Pardus wealth distribution to real world data

country	year	α	method	source
Pardus	2010	2.46	data from MMOG	own fit
Ancient Egypt	ca. 1380 BC	3.76 ± 0.22	excavation data	[13]
Hungary	1550	0.92	historical almanac	[14]
Hungary	1767 – 1773	0.99	historical almanac	[14]
Sweden	1931	1.5	wealth tax	[18]
Sweden	1959	1.7	wealth tax	[18]
India	1991	2.04 – 2.44	survey	[11]
India	2002	1.85 – 2.17	survey	[11]
France	1994	1.82 ± 0.03	encyclopedia	[19]
UK	1996	1.9	inheritance tax	[12]
UK	1997	1.06 ± 0.004	ranking of wealthiest	[19]
Sweden	1999	1.54 ± 0.05	wealth tax	own fit, see Fig. S1
Sweden	2000	1.58 ± 0.02	wealth tax	own fit, see Fig. S1
Sweden	2001	1.64 ± 0.02	wealth tax	own fit, see Fig. S1
Sweden	2002	1.61 ± 0.04	wealth tax	own fit, see Fig. S1
Sweden	2003	1.61 ± 0.03	wealth tax	own fit, see Fig. S1
Sweden	2004	1.61 ± 0.04	wealth tax	own fit, see Fig. S1
Sweden	2005	1.62 ± 0.04	wealth tax	own fit, see Fig. S1
Sweden	2006	1.59 ± 0.05	wealth tax	own fit, see Fig. S1
Sweden	2007	1.63 ± 0.04	wealth tax	own fit
USA	1988 – 2003	1.1 – 1.7	ranking of wealthiest	[15]
India	2002	0.81	ranking of wealthiest	[16]
India	2004	0.92	ranking of wealthiest	[16]
China	2003	2.285	ranking of wealthiest	[17]
China	2004	2.043	ranking of wealthiest	[17]
China	2005	1.758	ranking of wealthiest	[17]
