# Supplementary material for: Behavioral and Network Origins of Wealth Inequality: Insights from a Virtual World
Source: PLoS One. 2014 Aug 25;9(8):e103503. doi: 10.1371/journal.pone.0103503 (PMC4143195; doi:10.1371/journal.pone.0103503)
Supplement: Table S2 — Linear regression model for wealth. Data taken at days 240, 480, 720, 960, and 1200 after the beginning of the game. . (PDF) [file pone.0103503.s006.pdf]

Table S2. Linear regression model for wealth.

	day 240	day 480	day 720	day 960	day 1200
constant	9.3×10^5	-7.61×10^6	-1.63×10^7	-1.06×10^7	-1.49×10^7
age	$-6.52 \times 10^{3**}$	$-9.73 \times 10^{3***}$	$-9.41 \times 10^{3***}$	$-1.15 \times 10^{4****}$	$-1.19 \times 10^{4***}$
activity a	5.57^{****}	7.87^{****}	9.67^{****}	10.2^{****}	13.1^{****}
faction rank	$5.44 \times 10^{5****}$	$3.56 \times 10^{5***}$	$4.25 \times 10^{5***}$	$6.21 \times 10^{5***}$	$6.28 \times 10^{5**}$
XP	33.7^{****}	12.9^{****}	3.41	0.139	-1.49
combat skill	$-1.32 \times 10^{5****}$	1.66×10^4	-3.74×10^4	-2.32×10^4	$-3.44 \times 10^{5***}$
farming skill	$1.47 \times 10^{5**}$	$4.4 \times 10^{5****}$	$6.44 \times 10^{5****}$	$8.47 \times 10^{5****}$	$1.68 \times 10^{6****}$
f_{trade}	1.65×10^6	1.75×10^6	-2.4×10^6	4.35×10^6	6.89×10^6
f_{messages}	1.27×10^6	-4.41×10^5	-2.96×10^6	-4.86×10^6	3.54×10^6
f_{attacks}	-2.01×10^6	-2.6×10^6	8.61×10^6	-7.59×10^6	-2.03×10^7
f_{good}	-2.67×10^6	2.74×10^5	1.03×10^7	-2.9×10^6	-9.44×10^6
$k_{\text{in}}^{\text{trade}}$	$4.6 \times 10^{4****}$	$1.37 \times 10^{5****}$	$2.07 \times 10^{5****}$	$2.75 \times 10^{5****}$	$4.71 \times 10^{5****}$
$k_{\text{out}}^{\text{trade}}$	$-2.49 \times 10^{4****}$	$-3.7 \times 10^{4**}$	$-5.27 \times 10^{4***}$	$-6.96 \times 10^{4**}$	-5.59×10^3
C^{trade}	-7.87×10^4	8.44×10^5	5.38×10^5	-2.42×10^6	1.79×10^6
$k_{\text{nn}}^{\text{trade}}$	$-1.01 \times 10^{4***}$	$-1.62 \times 10^{4*}$	$-2.96 \times 10^{4*}$	$-4.18 \times 10^{4*}$	$-8 \times 10^{4**}$
$k_{\text{in}}^{\text{comm.}}$	$8.32 \times 10^{4**}$	$9.51 \times 10^{4*}$	7.51×10^4	$-1.76 \times 10^{5*}$	3.23×10^5
$k_{\text{out}}^{\text{comm.}}$	$-6.63 \times 10^{4***}$	$-9.38 \times 10^{4*}$	-3.14×10^4	$1.66 \times 10^{5*}$	-1.44×10^5
$C^{\text{comm.}}$	$-1.01 \times 10^{6*}$	-7.99×10^5	-1.89×10^6	-1.3×10^6	-1.34×10^6
$k_{\text{nn}}^{\text{comm.}}$	-3.62×10^3	$-1.38 \times 10^{4*}$	3.05×10^3	7.2×10^3	-1.33×10^4
$k_{\text{in}}^{\text{friend}}$	-9.37×10^3	6.91×10^4	1.87×10^3	1.33×10^5	-1.12×10^4
$k_{\text{out}}^{\text{friend}}$	1.14×10^4	$-7.51 \times 10^{4*}$	$-1.64 \times 10^{5***}$	$-1.85 \times 10^{5***}$	$-3.16 \times 10^{5****}$
C^{friend}	$1.49 \times 10^{6***}$	1.2×10^6	4.76×10^5	2.99×10^5	8.69×10^5
$k_{\text{nn}}^{\text{friend}}$	$-1.78 \times 10^{4*}$	-1.87×10^4	-2.14×10^4	-1.07×10^4	-2.01×10^4
$k_{\text{in}}^{\text{enemy}}$	3.04×10^3	-6.86×10^3	-2.59×10^4	-8.46×10^3	$1.18 \times 10^{5***}$
$k_{\text{out}}^{\text{enemy}}$	$-3.51 \times 10^{4*}$	$-7.31 \times 10^{4**}$	-4.28×10^4	$-1.28 \times 10^{5**}$	$-1.76 \times 10^{5**}$
C^{enemy}	5.08×10^5	-3.84×10^6	-4.25×10^6	-7.47×10^6	1.82×10^7
$k_{\text{nn}}^{\text{enemy}}$	$4.18 \times 10^{3*}$	4.04×10^3	7.03×10^3	$2.45 \times 10^{4**}$	2.91×10^3
	$r^2 = 0.41793$	$r^2 = 0.39313$	$r^2 = 0.3883$	$r^2 = 0.38314$	$r^2 = 0.39135$

Data taken at days 240, 480, 720, 960, and 1200 after the beginning of the game.
* $p -$ value < 0.05, ** $p -$ value < 0.01, *** $p -$ value < 0.001, **** $p -$ value < 0.0001.
