# Supplementary material for: Behavioral and Network Origins of Wealth Inequality: Insights from a Virtual World
Source: PLoS One. 2014 Aug 25;9(8):e103503. doi: 10.1371/journal.pone.0103503 (PMC4143195; doi:10.1371/journal.pone.0103503)
Supplement: Text S1 — Definition of Network Properties. (PDF) [file pone.0103503.s008.pdf]

Text S1. Definition of Network Properties.

S1.1 Directed and Undirected Networks

A network $\mathcal{G}$, called graph in mathematics, consists of a set $\mathcal{N}$ of *nodes* n_i and a set $\mathcal{L}$ of *links* connecting these nodes: $\mathcal{G} := (\mathcal{N}, \mathcal{L})$ [1–3]. In a directed network, links are ordered pairs: $\mathcal{L} \ni l_{ij} := (n_i, n_j)$ is a link from node n_i to node n_j. In an undirected network, links are unordered pairs of nodes: $\mathcal{L}_{\text{undir}} \ni l_{ij} := \{n_i, n_j\}$. Here, a node represents a player in the game, while a link represents an interaction between two players. For each of the interaction types we studied, we produce a separate network, denoted by a superscript on the associated quantities. The (directed) links are constructed in the following way:

$l_{ij} \in \mathcal{L}^{\text{trade}}$ if player i has traded with a building of player j,

$l_{ij} \in \mathcal{L}^{\text{comm.}}$ if a message has been sent from player i to player j,

$l_{ij} \in \mathcal{L}^{\text{friend}}$ if player i has marked player j as friend,

$l_{ij} \in \mathcal{L}^{\text{enemy}}$ if player i has marked player j as enemy.

The *symmetrization* $\mathcal{G}_{\text{undir}} = (\mathcal{N}, \mathcal{L}_{\text{undir}})$ of a directed network $\mathcal{G} = (\mathcal{N}, \mathcal{L})$ is constructed in the following way: Starting with $\mathcal{G}_{\text{undir},0} = (\mathcal{N}, \mathcal{L}_{\text{undir},0})$, where $\mathcal{L}_{\text{undir},0} = \emptyset$, a link l_{ij} is added to $\mathcal{L}_{\text{undir},0}$ if $l_{ij} \in \mathcal{L}$ or if $l_{ji} \in \mathcal{L}$.

S1.2 Degree

In an undirected network, the *degree* $k_{\text{undir},i}$ of n_i is the number of other nodes n_j, for which the link l_{ij} is present in the network, $k_{\text{undir},i} := \#\{n_j : l_{ij} \in \mathcal{L}_{\text{undir}}\}$ (where $\#\{\dots\}$ means the cardinality, i.e. the number of elements of the set). $\mathcal{N}_i := \{n_j \in \mathcal{N} : l_{ij} \in \mathcal{L}_{\text{undir}}\}$ is called the set of (nearest) *neighbors* of node n_i. In a directed network, there are two degrees: The *indegree* $k_{\text{in},i}$ of node n_i is the number of other nodes n_j, from which a link points to node n_i in the network, $k_{\text{in},i} := \#\{n_j : l_{ji} \in \mathcal{L}\}$. Accordingly, the *outdegree* $k_{\text{out},i}$ of node n_i is the number of other nodes n_j, to which a link points from node n_i in the network, $k_{\text{out},i} := \#\{n_j : l_{ij} \in \mathcal{L}\}$.

S1.3 Clustering Coefficient

In an undirected network, the *clustering coefficient* C_i of node n_i is the ratio of pairs of neighbors of n_i that are connected to the number of all pairs of neighbors of n_i,

$$C_i := \frac{\#\{l_{jk} \in \mathcal{L} : n_j \in \mathcal{N}_i \wedge n_k \in \mathcal{N}_i\}}{\frac{1}{2}k_i(k_i - 1)} \quad .$$

Clustering coefficients have been calculated using the function '`clustercoeffs`' from the package 'gaimc' by David Gleich[1].

S1.4 Nearest-Neighbor Degree

In an undirected network, the *nearest-neighbor degree* $k_{\text{nn},i}$ of node n_i is the average (undirected) degree of its neighbors,

$$k_{\text{nn},i} := \langle k_{\text{undir},j} \rangle_{n_j \in \mathcal{N}_i} \quad .$$

[1] available at `https://github.com/dgleich/gaimc`

References

1. Wasserman S, Faust K (1994) Social Network Analysis: Methods and Applications. Cambridge University Press.

2. Dorogovtsev S, Mendes J (2003) Evolution of Networks: From Biological Nets to the Internet and WWW. Oxford: Oxford University Press.

3. Newman MEJ (2010) Networks: an Introduction. Oxford: Oxford University Press.
